# Adolescents and Young Adults Evaluating a Website for Affective-Sexual Information and Education: Multicenter Cross-Sectional Study

**DOI:** 10.2196/49962

**Published:** 2023-10-26

**Authors:** Laura Montero-Pons, Dolors Rodríguez-Martín, Cristina Esquinas, Rosa García-Sierra, Josep Maria Manresa-Domínguez, Azahara Reyes-Lacalle, Rosa Cabedo-Ferreiro, MªMercedes Vicente-Hernández, Míriam Gómez Masvidal, Pere Toran-Monserrat, Gemma Falguera-Puig

**Affiliations:** 1 Sexual and Reproductive Healthcare Santa Coloma de Gramenet Primary Care Management Metropolitana Nord Catalan Institute of Health Santa Coloma de Gramenet Spain; 2 Consolidated Research Group on Sexual and Reproductive Healthcare Barcelona Spain; 3 Faculty of Nursing Universitat de Barcelona L’Hospitalet de Llobregat Spain; 4 Department of Basic and Clinical Nursing Faculty of Nursing Universitat de Barcelona L’Hospitalet de Llobregat Spain; 5 Consolidated Research Group on Gender, Identity and Diversity Barcelona Spain; 6 Interuniversity Research Group on Gender, Diversity and health Barcelona Spain; 7 Department of Public Health, Mental Health and Maternal and Child Health Faculty of Nursing Universitat de Barcelona L’Hospitalet de Llobregat Spain; 8 Multidisciplinary Research Group in Health and Society Barcelona Spain; 9 Research Support Unit Metropolitana Nord Primary care Research Institut Universitari d’Investigació en Atenció Primària Jordi Gol Mataró Spain; 10 Nursing Department Faculty of Medicine Universitat Autònoma de Barcelona Barcelona Spain; 11 Primary Care Group Germans Trias i Pujol Research Institute Badalona Spain; 12 Sexual and Reproductive Healthcare Sabadell Primary Care Management Metropolitana Nord Catalan Institute of Health Sabadell Spain; 13 Sexual and Reproductive Healthcare Granollers Primary Care Management Metropolitana Nord Catalan Institute of Health Granollers Spain; 14 Sexual and Reproductive Healthcare Sant Adrià del Besòs Primary Care Management Metropolitana Nord Catalan Institute of Health Sant Adrià del Besòs Spain; 15 Sexual and Reproductive Healthcare Mataró/Maresme Primary Care Management Metropolitana Nord Catalan Institute of Health Mataró Spain; 16 Department of Medicine Faculty of Medicine Universitat de Girona Girona Spain; 17 Sexual and Reproductive Healthcare Directorate Primary Care Management Metropolitana Nord Catalan Institute of Health Sabadell Spain

**Keywords:** sex education, adolescent, young adult, internet, cross-sectional studies, program evaluation, gender mainstreaming

## Abstract

**Background:**

Today’s young people have long been demanding a paradigm shift in the emotional and sexual education they receive. While for them, affective-sexual and gender diversity is already a reality, the sexual and reproductive health professionals they encounter lack sufficient training. The digital devices and affective-sexual education websites aimed at today’s young people must also be thoroughly evaluated. The website Sexe Joves is a website on sexuality by the Department of Health of the Government of Catalonia (Spain). It is designed for people aged 14 to 25 years. It currently needs to undergo a process of evaluation. Affective-sexual education aimed at young people must stem from their participation and the whole range of sexual and gender diversity in order to reach the entire population equally.

**Objective:**

The aim of this study was to evaluate the website Sexe Joves as a source of affective-sexual health information, education, and communication for young people. It takes into account sex, gender identity, sexual orientation, socioeconomic status, and location within Catalonia (urban, semiurban, and rural areas).

**Methods:**

This was an observational, descriptive, and cross-sectional study that forms part of a larger mixed methods study. An ad hoc questionnaire was used to collect data. In total, 1830 participants were included. The study was carried out simultaneously in all the territorial administrations of Catalonia.

**Results:**

Almost 30% of the sample obtained were young people who experience affective-sexual and gender diversity. Of those surveyed, only 14.2% (n=260) said they were familiar with the website and of these, 6.5% said they used it (n=114). The website content rated most indispensable was on sexual abuse, harassment, and violence, followed by sexually transmitted infections; 70.5% (n=1200) reported that they visit pornographic websites.

**Conclusions:**

The results of this study will contribute to the design of new strategies for the website Sexe Joves, a public health resource, in order to improve affective sexual education for young people.

**International Registered Report Identifier (IRRID):**

RR2-10.3390/ijerph192416586

## Introduction

### Background

There are three fundamental pillars that justify the need for this study: (1) the paradigm shift that young people demand in terms of the affective-sexual education they receive, (2) the failure to keep sexual and reproductive health care professionals up-to-date on the reality of the affective-sexual and gender diversity (ASGD) that these young people experience, and (3) the increase in gender-based violence against women treated by health services [1,2].

Young people today claim they have received sex education too late and many have already had sexual encounters. They generally define it as a coitocentric, biomedical, heteropatriarchal, and binary model [3]. This paradigm of sexual education is based on fear and prioritizes preventing unplanned pregnancies and sexually transmitted infections (STIs). It essentially only takes into account sex focused on penetration. The lack of affective-sexual education that promotes positive sexuality based on self-esteem, self-care, and self-knowledge, as well as respect and empathy [3], together with the ease of accessing mainstream pornography—which young people often accidentally arrive at when searching for other topics on the internet—lead to a lack of healthy references for young people with regard to their sexuality [2,4].

The lesbian, gay, bisexual, trans, intersex community has often expressed its upset at the treatment received in health services [2]. Sexual and reproductive health care professionals must receive continuous training to stay up-to-date in terms of their knowledge of and attitudes toward ASGD [1].

Gender-based violence is a serious public health problem and a violation of human rights [2,5]. It includes sexual violence, or the attempt to obtain sexual acts without the other’s consent, which, in turn, encompasses sexual harassment, sexual abuse, exhibition, and the imposition of any sexual practice or assault, including rape [2].

The website Sexe Joves (WSJ) on sexuality is a reference for health professionals in Catalonia [6]. The project started in 2003 and was fully operative in 2006. It was led by midwives from the outset, with support from the government of Catalonia and the Catalan Institute of Health (Institut Català de la Salut; ICS). A multidisciplinary team of 65 professionals from various institutions related to sexuality and youth participated in the creation process. This group prepared a document with content for the website, which was validated by a total of 153 young people from secondary schools across Catalonia [7]. The main aims are twofold: (1) to help young people enjoy a healthy and responsible sexuality, and (2) to prevent unplanned pregnancies and STIs. The website offers information on affection, self-esteem, knowledge about one’s body, abuse, harassment, sexual assault, sex and drugs, and cybersex. It also includes a virtual consultation room (email and chats), where a team of midwives provides individualized care and links to health centers that offer face-to-face or telephone consultations [3]. The website is designed for 14- to 25-year-olds. The advice takes into account sex, sexual orientation, and gender identity [8]. The WSJ has a multidisciplinary editorial committee, which consists of 9 midwives, 2 psychologists, a gynecologist, an administrator, and 2 senior public health technicians from the Catalan government, and it is coordinated by the midwife who launched the project. The committee is responsible for the day-to-day running of the website, updating its content, and analyzing indicators such as number of visits, telematic consultations, and most consulted topics [8].

Affective-sexual health education for young people in Catalonia is provided by the public universal primary care system, specifically from the Reproductive and Sexual Health Care units (ASSIR, the abbreviation for the Catalan name, Atenció a la Salut Sexual i Reproductiva) [9,10].

Although the website has been operative for 16 years, it was only evaluated in 2012 as part of a doctoral dissertation [7]. Through a quantitative study with a descriptive design that analyzed several variables from the website, as well as the user satisfaction of young people, it was concluded that at that time, the WSJ had a high usability rate (in a website or computer program refers to ease of use, taking into account readability of texts, speed of download, manageability, and capacity to meet the needs of the user [11]). In the second phase of the same doctoral dissertation, an analysis was conducted to evaluate the quality of responses from the midwives with a validated questionnaire. In the third phase, a quasi-experimental study was performed, pre- and posttraining, of the midwives in charge of responding to the website’s virtual consultation. The dissertation concluded that the quality of responses to emails was high and that up-to-date training of professionals who answer these emails would be sufficient to reduce inadequate responses [7]. The editorial committee has now commissioned the research team of this project to undertake an in-depth evaluation of the website centered around the voice of the youth of Catalonia [3]. After a systematic review, the need to conduct a mixed methods study became clear since, on the one hand, the editorial committee requested that an in-depth design be developed with the participation of the website users, young people, to understand their discourses. On the other hand, only a quantitative study had been carried out to assess the sexual health website preferences of adolescents in Germany [12]. A quantitative study was deemed appropriate since it could provide insight into the preferences of a wider sample and highlight and quantify differences between different subpopulations. The results of this study will contribute essential data to determine new strategies for the sustainability and relevance of the WSJ. The results might also provide advice for professionals in primary health care, school health, and community nursing. The study will outline a new participatory model of affective sexual education for adolescents and young adults, led by midwives and nurses, and which incorporates gender mainstreaming and socioeconomic diversity.

### Researchers’ Description

The principal investigator and the majority of researchers are midwives working at the ASSIR units, where they offer affective sexual education to young people during individual visits, as well as to the community at education centers and various associations and public spaces. They are also linked to primary care research. The research team includes research staff and university professors, as well.

Following the recommendations of Chapman et al [13], it should be noted that among the research staff and midwives who collected samples, 90% had a female gender identity and 10% had a male gender identity.

### General Objective of the Project

The aim of this study was to evaluate the WSJ as a source of affective-sexual health information, education, and communication for young people based on a holistic perspective of affective sexual health that includes (1) sexual and gender diversity and (2) socioeconomic factors. This study forms part of a larger mixed methods project titled “Adolescents and Young Adults Evaluating a Website for Affective-Sexual Information and Education in Catalonia.” To our knowledge, this is the first study to thoroughly explore the type of sex education demanded by the youth today in Spain [3].

## Methods

### Design

This is an observational, descriptive, cross-sectional quantitative study for which an ad hoc questionnaire was created.

This study adhered to the Strengthening the Reporting of Observational Studies in Epidemiology (STROBE) guidelines [14] and the Tool to Incorporate Gender Mainstreaming in Research by the Comunitat Hipàtia and promoted by the Agency of Health Quality and Assessment of Catalonia (AQUAS) [15].

The study protocol was approved in July 2020. The study lasted 2 years and participants were young people living anywhere in Catalonia, including urban, semiurban, and rural areas [16]. The fieldwork was conducted over 18 months.

The Department of Health of the Generalitat de Catalunya is organized into 9 territorial departments. In this study, a sample of all 9 territorial managements has been collected.

### Sample or Participants

The target population of the study was young people in Catalonia (Spain) between 14 and 25 years of age. This population comprises 972,604 people in Catalonia and 6,147,560 in Spain. Participants were teenagers and young adults aged 14 to 25 years able to express themselves in 1 of the 2 official languages of Catalonia (Catalan or Spanish). We excluded people who did not wish to participate in the study and people under 16 who wished to participate but whose parents or legal guardians did not sign the informed consent form.

We devised three methods for participant recruitment: (1) convenience sampling at education centers (high schools, vocational training schools, training and insertion programs, and universities), (2) consecutive sampling to recruit young people who visited the ASSIR units, and (3) young people who accessed the WSJ of their own accord were invited to participate. These 3 methods were used to invite participants to join the study, and participation was voluntary. Recruitment is more fully developed in the data collection Section.

### Data Collection

#### Overview

In the context of COVID-19, we had to have both web-based (Microsoft Forms) and paper versions of the questionnaire (Teleform). The web-based questionnaire could be accessed via a direct link or QR code. The questionnaire consisted of 24 closed questions and 2 open-ended questions. The questionnaire is available in Multimedia Appendix 1. At the education centers, the recruiting researchers communicated with these centers to disseminate the questionnaire among students. The center directors decided if their center would use a web-based or paper questionnaire. At the ASSIR units, a wide network of professionals who were also researchers recruited for the study. The study was disseminated through posters in the waiting rooms and at the counter of each ASSIR unit. These posters contained the study instructions and the QR code for the questionnaire so that young people could quickly download it to their mobile phones and answer it. There was a paper version of the questionnaire for people who could not access it digitally. Digital users of the ASSIR were sent the link to the questionnaire with information to encourage them to answer on the same platform they used for the consultation (email, WhatsApp Messenger by Meta Platforms, and e-consultation). WSJ users who made email inquiries automatically received an email with a direct link to the questionnaire, encouraging them to participate after explaining that it was a study to evaluate the website. The study was also advertised on the WSJ homepage through images with a direct link to the questionnaire.

Whatever the origin of the participant, the following variables were evaluated for all the participants in the sample through the questionnaire:

#### Sociodemographic Variables

Age, sex, gender identity, sexual orientation, place of origin, born in Spain or abroad, city/rural/semirural residence, socioeconomic condition according to the Territorial Socioeconomic Index (TSI), type of access to the questionnaire (electronic or paper), current job and ongoing studies*.*

To establish the participant’s culture of origin, the mother’s place of origin was used, as this tends to be the main transmitter of cultural identity, through gender roles and stereotypes [17,18].

The TSI was used to determine socioeconomic conditions. This is a synthetic index that summarizes in a single value several socioeconomic characteristics of the population by small areas. The index includes data on employment, educational level, immigration, and income of all people living in each territorial unit, based on 6 sectoral indicators [19].

In order to associate each municipality with the corresponding degree of urbanization—urban, semiurban, and rural areas—we requested a database with this information from the Barcelona Institute of Regional and Metropolitan Studies, who provided data for all of Catalonia.

#### Variables Related to the WSJ

Prior knowledge of the WSJ, use of the WSJ, usability of the WSJ, usefulness of the WSJ, and rating of the affective sexual education content of the WSJ, including the topics (1) emotion and sexuality; (2) knowledge of your body; (3) petting; (4) the first time; (5) contraception; (6) emergency contraception; (7) pregnancy; (8) abortion; (9) STIs; (10) abuse, harassment, and sexual violence; (11) sex and drugs; (12) cybersex; and (13) cyberbullying.

#### Variables Related to Social Media

We measured the ability to access digital resources, use of other webpages for affective-sexual education, whether the participant followed affective-sexual education influencers, and use of pornography webpages.

#### Variables Related to Study Recruitment

Participants from educational centers and ASSIR units accessed the survey by indication of the professionals. The third contacting group accessed it by themselves, without anyone’s indication. The sample calculation was performed based on this variable.

### Ethics Approval

Data collection for this study coincided with the COVID-19 pandemic during times of significant restrictions in Catalonia, such that the measures explained in this section had to be taken to continue implementing the project. These changes were accepted by the research ethics committee.

Participants had to give their informed written consent. Participants under the age of 16 years also had to present the consent of their parents or legal guardians. This is known as double consent. Participants aged 16 to 18 years were considered to be mature minors and did not need the consent of their parents or legal guardians. The questionnaires were anonymous and voluntary.

This protocol follows the tenets of the Declaration of Helsinki [20]. The research protocol was approved by the research ethics committee of the Institut d’Investigació en Atenció Primària Jordi Gol in July 2020 (19/074-P). It was also approved by the management of ASSIR Catalunya and the editorial committee of the WSJ. All the directors of the education centers where the study was conducted were adequately informed.

Due to COVID-19, Microsoft Forms software had to be used. In this case, we guaranteed data protection, in accordance with the Regulation (European Union) 2016/679 of the European Parliament and Council of April 27 on Data Protection (General Data Protection Regulation) and the Spanish Organic Law 3/2018 of December 5, on the Protection of Personal Data and guarantee of digital rights. The web-based questionnaire could only be answered by people aged 16 years or older since the necessary consent could not be obtained from children under 16. Consequently, the first question in the web-based questionnaire was if they agreed to answer it, and the second was if they were 16 or older. Responses from those under 16 were collected using the paper questionnaire at the education centers and ASSIR units.

### Statistical Analysis

Qualitative variables were described with absolute frequencies and percentages. Quantitative variables were described using the mean and SD. Items included in the WSJ were analyzed according to the sociodemographic variables of interest. The chi-squared test (Fisher test for frequencies <5) was used for these comparisons. For all the tests, *P* values <.05 were considered statistically significant. The statistical package R Studio (version 2.5.1; Posit) was used for the analyses.

## Results

### Sociodemographic Variables

The sample was composed of 1830 young people. Regarding biological sex (manifestation of sex through external genitalia) [21], 23.8% (n=434) were male, 75.4% (n=1373) were female, and 0.8% (n=15) were intersex. As for gender identity, 72.4% (n=1262) identified as women, 24.8% (n=432) as men, 2.3% (n=40) as nonbinary, and 0.5% (n=9) as other. Regarding sexual orientation, 72.6% (n=1304) described themselves as heterosexual, 21.9% (n=393) as bisexual, 3.4% (n=61) as homosexual, and 2.1% (n=37) as other (Table 1).

As for cultural origin, 84.7% (n=1413) of participants came from Spain, 10.7% (n=179) from Latin American countries, 1.6% (n=27) from other European countries, 1.5% (n=25) from North Africa, 0.7% (n=12) from sub-Saharan African countries, and 0.7% (n=12) from Asia and Oceania. As for place of birth, 86.9% (n=1590) of participants were born in Spain and 13.1% (n=239) were born abroad.

A total of 24.6% (n=451) of participants did not have the digital resources to answer the web-based questionnaire and had to complete it on paper (ie, they were experiencing digital inequality); 32.7% (n=599) of the young people in the sample had a very low TSI (Table 1).

Regarding employment, 67.2% (n=1199) were unemployed, while 32.8% (n=585) were employed (Table 1).

**Table 1:** Sociodemographic variables (N=1830).

Variable			Participants, n (%)
**Age (years) (n=1830^a^)**			
	14-18	1033 (56.4)	
	19-26	797 (43.6)	
**Sex (n=182^a^)**			
	Male	434 (23.8)	
	Female	1373 (75.4)	
	Intersex	15 (0.8)	
**Sexual orientation (n=1795^a^)**			
	Heterosexual	1304 (72.6)	
	Bisexual	393 (21.9)	
	Homosexual	61 (3.4)	
	Other	37 (2.1)	
**Gender identity (n=1743^a^)**			
	Man	432 (24.8)	
	Woman	1262 (72.4)	
	Nonbinary	40 (2.3)	
	Other	9 (0.5)	
**Place of residence (n=1814^a^)**			
	Urban	1471 (81.1)	
	Semiurban	213 (11.7)	
	Rural	130 (7.2)	
**Territorial Socioeconomic Index (n=1827^a^)**			
	Very low socioeconomic level	599 (32.7)	
	Low socioeconomic level	460 (25.2)	
	High socioeconomic level	646 (35.4)	
	Very high socioeconomic level	122 (6.7)	
**Ongoing studies (n=1816^a^)**			
	I do not study	167 (9.2)	
	Secondary school	195 (10.7)	
	High school	652 (35.9)	
	Lower-level vocational training	173 (9.5)	
	Higher-level vocational training	170 (9.4)	
	Training and insertion programs	33 (1.8)	
	University	426 (23.5)	
**Currently employed (n=1784^a^)**			
	No	1199 (67.2)	
	Yes	585 (32.8)	

^a^Total number of respondents for each variable.

### Variables Related to the WSJ

Regarding familiarity with the WSJ, 14.2% (n=260) of respondents reported already knowing of it and 6.5% (n=114) said they used it. A total of 90% (n=1634) did not believe that other young people in their setting were familiar with it. Of those who were familiar with and used the WSJ, 92.9% (n=247) did so via their mobiles, while 14.3% (n=38) visited it from a computer. Regarding content accessibility, 23.2% (n=98) stated that it is not easy to find the topic they’re searching for. Nonetheless, 76.3% (n=274) of participants who were already familiar with the WSJ believed it was useful. The WSJ chat was used by 43.3% (n=161) of participants while 24.7% (n=92) emailed their questions (Table 2).

Of the affective sexual education content on the WSJ, it should be noted that the topic that was rated most indispensable was sexual abuse, harassment, and violence, at 70.6% (n=1246), followed by STIs, at 66% (n=1167). Aspects such as contraception, emergency contraception, and pregnancy had scores below 45%, much like the topic of sex and drugs (n=1762, 40.4%). By contrast, abortion did receive a score above 45% (n=955, 54.2%).

There were significant differences in how young adults and adolescents rated the content indispensable, specifically with regard to emotion and sexuality (32.2% vs 46.6%), contraception (46.8% vs 38%), and cybersex (17% vs 12%; all *P*<.05; Table 3). As for biological sex, significant differences were identified regarding cybersex content for intersex people, who gave it an indispensability rating of 57.1% (8/14), while male and female participants gave it a score of just 11.1% (47/424) and 14.7% (193/1315), respectively. Regarding gender identity, it is worth noting differences in cyberbullying content for those who identified as nonbinary (25/40, 62.5%) and those who identified as men (174/420, 41.4%). In terms of sexual orientation, significant differences were found across all scores (Table 3). There was no significant difference in how the young people of Catalonia rated the contents of the WSJ based on whether they lived in urban, semiurban, or rural areas. There were significant differences depending on the education level of the participants. For example, university students gave STIs a score of 73.6% (310/421), while participants in training and insertion programs gave it 43.8% (14/32, *P*=.001). Last, regarding whether or not participants were employed, very significant differences were identified—(all *P*<.001)—in the scores of employed versus unemployed participants for the topics of contraception and sexual abuse, harassment, and violence. There were also significant differences in the evaluation of the contents of the WSJ, depending on whether the participants were born in Spain or not (Table 4).

It is interesting how the highest percentage of participants who knew the WSJ, according to their TSI, were those with a very low TSI (20.1%). On the other hand, there were no significant differences regarding the evaluation of the contents according to the TSI of the participants, with the exception of cyberbullying. This content is better valued by the high levels of TSI (92.1% vs 85.4%).

**Table 2:** Variables related to the WSJ^a^ and social media (N=1830).

Variable		Participants, n (%)
**Prior knowledge of the WSJ (n=1826^b^)**		
	No	1566 (85.8)
	Yes	260 (14.2)
**Use of the WSJ (n=1763^b^)**		
	No	1649 (93.5)
	Yes	114 (6.5)
**Usability of the WSJ (n=423^b^)**		
	No	98 (23.2)
	Yes	325 (76.8)
**Usefulness of the WSJ (n=359^b^)**		
	No	85 (23.7)
	Yes	274 (76.3)
**Use for the content of the WSJ (n=372^b^)**		
	No	164 (44.1)
	Yes	208 (55.9)
**Use of the WSJ chat (n=372^b^)**		
	No	211 (56.7)
	Yes	161 (43.3)
**Use of the WSJ via email (n=372^b^)**		
	No	280 (75.3)
	Yes	92 (24.7)
**Do you visit other websites? (n=1694^b^)**		
	No	1537 (90.7)
	Yes	157 (9.3)
**Follow influencers (n=1695^b^)**		
	No	1322 (78)
	Yes	373 (22)
**Pornography (n=1701^b^)**		
	No	501 (29.5)
	Yes	1200 (70.5)

^a^WSJ: Website Sexe Joves.

^b^Total number of responses for each variable.

**Table 3:** Rating of affective sexual education content of the website Sexe Joves related to participants’ age and sexual orientation (N=1830).

Variable			Respondents, n/N (%)		Respondents aged 14-18 years (n=1033), n/N (%)		Respondents aged 19-26 years (n=1033), n/N (%)		*P* value			Heterosexual respondents (n=1304), n/N (%)			Bisexual respondents (n=393), n/N (%)			Homosexual respondents (n=61), n/N (%)			Other (n=37), n/N (%)			*P* value^a^	
**Emotion and sexuality**										<.001															<.001
	Unimportant	107/1761 (6.1)		84/999 (8.4)		23/762 (3)					84/1253 (6.7)			12/385 (3.1)			2/60 (3.3)			5/37 (13.5)					
	Important	975/1761 (55.4)		591/999 (59.2)		384/762 (50.4)					725/1253 (57.9)			184/385 (47.8)			26/60 (43.3)			23/37 (62.2)					
	Indispensable	679/1761 (38.6)		324/999 (32.4)		355/762 (46.6)					444/1253 (35.4)			189/385 (49.1)			32/60 (53.3)			9/37 (24.3)					
**Knowledge of your body**										.05															<.001
	Unimportant	38/1771 (2.1)		21/1005 (2.1)		17/766 (2.2)					31/1260 (2.5)			2/386 (0.5)			0/60 (0)			3/37 (8.1)					
	Important	610/1771 (34.4)		370/1005 (36.8)		240/766 (31.3)					470/1260 (37.3)			90/386 (23.3)			19/60 (31.7)			14/37 (37.8)					
	Indispensable	1123/1771 (63.4)		614/1005 (61.1)		509/766 (66.4)					759/1260 (60.2)			294/386 (76.2)			41/60 (68.3)			20/37 (54.1)					
**Petting**										.25															.001
	Unimportant	617/1742 (35.4)		335/990 (33.8)		282/752 (37.5)					469/1237 (37.9)			104/381 (27.3)			20/60 (33.3)			13/37 (35.1)					
	Important	888/1742 (51)		520/990 (52.5)		368/752 (48.9)					624/1237 (50.4)			207/381 (54.3)			28/60 (46.7)			17/37 (45.9)					
	Indispensable	237/1742 (13.6)		135/990 (13.6)		102/752 (13.6)					144/1237 (11.6)			70/381 (18.4)			12/60 (20)			7/37 (18.9)					
**The first time**										.06															<.001
	Unimportant	333/1769 (18.8)		172/1003 (17.1)		161/766 (21)					194/1258 (15.4)			96/386 (24.9)			18/60 (30)			16/37 (43.2)					
	Important	1017/1769 (57.5)		579/1003 (57.7)		438/766 (57.2)					735/1258 (58.4)			220/386 (57)			31/60 (51.7)			14/37 (37.8)					
	Indispensable	419/1769 (23.7)		252/1003 (25.1)		167/766 (21.8)					329/1258 (26.2)			70/386 (18.1)			11/60 (18.3)			7/37 (18.9)					
**Contraception**										<.001															<.001
	Unimportant	165/1738 (9.5)		115/988 (11.6)		50/750 (6.7)					124/1232 (10.1)			19/383 (5)			11/60 (18.3)			5/37 (13.5)					
	Important	838/1738 (48.2)		489/988 (49.5)		349/750 (46.5)					608/1232 (49.4)			166/383 (43.3)			32/60 (53.3)			16/37 (43.2)					
	Indispensable	735/1738 (42.3)		384/988 (38.9)		351/750 (46.8)					500/1232 (40.6)			198/383 (51.7)			17/60 (28.3)			16/37 (43.2)					
**Emergency contraception**										.44															<.001
	Unimportant	136/1740 (7.8)		84/988 (8.5)		52/752 (6.9)					98/1235 (7.9)			15/383 (3.9)			11/60 (18.3)			5/37 (13.5)					
	Important	922/1740 (53)		523/988 (52.9)		399/752 (53.1)					668/1235 (54.1)			192/383 (50.1)			29/60 (48.3)			21/37 (56.8)					
	Indispensable	682/1740 (39.2)		381/988 (38.6)		301/752 (40)					469/1235 (38)			176/383 (46)			20/60 (33.3)			11/37 (29.7)					
**Pregnancy**										.30															<.001
	Unimportant	146/1766 (8.3)		91/1001 (9.1)		55/765 (7.2)					83/1255 (6.6)			39/386 (10.1)			11/60 (18.3)			9/37 (24.3)					
	Important	894/1766 (50.6)		508/1001 (50.7)		386/765 (50.5)					649/1255 (51.7)			189/386 (49)			25/60 (41.7)			17/37 (45.9)					
	Indispensable	726/1766 (41.1)		402/1001 (40.2)		324/765 (42.4)					523/1255 (41.7)			158/386 (40.9)			24/60 (40)			11/37 (29.7)					
**Abortion**										.29															<.001
	Unimportant	110/1762 (6.2)		70/1002 (7)		40/760 (5.3)					81/1254 (6.5)			12/385 (3.1)			7/60 (11.7)			6/37 (16.2)					
	Important	697/1762 (39.6)		398/1002 (39.7)		299/760 (39.3)					530/1254 (42.3)			121/385 (31.4)			19/60 (31.7)			13/37 (35.1)					
	Indispensable	955/1762 (54.2)		534/1002 (53.3)		421/760 (55.4)					643/1254 (51.3)			252/385 (65.5)			34/60 (56.7)			18/37 (48.6)					
**Sexually transmitted infections**										.003															.01
	Unimportant	43/1767 (2.4)		24/1004 (2.4)		19/763 (2.5)					34/1258 (2.7)			4/385 (1)			0/60 (0)			3/37 (8.1)					
	Important	557/1767 (31.5)		349/1004 (34.8)		208/763 (27.3)					405/1258 (32.2)			103/385 (26.8)			21/60 (35)			14/37 (37.8)					
	Indispensable	1167/1767 (66)		631/1004 (62.8)		536/763 (70.2)					819/1258 (65.1)			278/385 (72.2)			39/60 (65)			20/37 (54.1)					
**Sexual abuse, harassment, and violence**										<.001															<.001
	Unimportant	58/1765 (3.3)		34/1002 (3.4)		24/763 (3.1)					52/1255 (4.1)			3/386 (0.8)			0/60 (0)			1/37 (2.7)					
	Important	461/1765 (26.1)		300/1002 (29.9)		161/763 (21.1)					347/1255 (27.6)			74/386 (19.2)			14/60 (23.3)			14/37 (37.8)					
	Indispensable	1246/1765 (70.6)		668/1002 (66.7)		578/763 (75.8)					856/1255 (68.2)			309/386 (80.1)			46/60 (76.7)			22/37 (59.5)					
**Sex and drugs**										.01															<.001
	Unimportant	240/1762 (13.6)		152/999 (15.2)		88/763 (11.5)					191/1256 (15.2)			34/386 (8.8)			4/60 (6.7)			8/37 (21.6)					
	Important	810/1762 (46)		469/999 (46.9)		341/763 (44.7)					592/1256 (47.1)			166/386 (43)			25/60 (41.7)			15/37 (40.5)					
	Indispensable	712/1762 (40.4)		378/999 (37.8)		334/763 (43.8)					473/1256 (37.7)			186/386 (48.2)			31/60 (51.7)			14/37 (37.8)					
**Cybersex**										<.001															.004
	Unimportant	721/1760 (41)		461/999 (46.1)		260/761 (34.2)					524/1254 (41.8)			139/383 (36.3)			22/60 (36.7)			21/37 (56.8)					
	Important	789/1760 (44.8)		418/999 (41.8)		371/761 (48.8)					575/1254 (45.9)			170/383 (44.4)			26/60 (43.3)			10/37 (27)					
	Indispensable	250/1760 (14.2)		120/999 (12)		130/761 (17.1)					155/1254 (12.4)			74/383 (19.3)			12/60 (20)			6/37 (16.2)					
**Cyberbullying**										.03															<.001
	Unimportant	206/1750 (11.8)		131/992 (13.2)		75/758 (9.9)					162/1244 (13)			26/384 (6.8)			3/60 (5)			8/37 (21.6)					
	Important	747/1750 (42.7)		431/992 (43.4)		316/758 (41.7)					546/1244 (43.9)			151/384 (39.3)			19/60 (31.7)			17/37 (45.9)					
	Indispensable	797/1750 (45.5)		430/992 (43.3)		367/758 (48.4)					536/1244 (43.1)			207/384 (53.9)			38/60 (63.3)			12/37 (32.4)					

^a^Calculated from chi-squared tests.

**Table 4:** Evaluation of the affective sexual education content of the website Sexe Joves in association with migration (N=1830).

Variable		Respondents, n/N (%)	Respondents born in Spain (n=1590), n/N (%)	Respondents born elsewhere (n=239), n/N (%)	*P* value^a^	
**Emotion and sexuality**						<.001
	Unimportant	107/1760 (6.1)	85/1540 (5.5)	22/220 (10)		
	Important	974/1760 (55.3)	838/1540 (54.4)	136/220 (61.8)		
	Indispensable	679/1760 (38.6)	617/1540 (40.1)	62/220 (28.2)		
**Knowledge of your body**						<.001
	Unimportant	38/1770 (2.1)	28/1544 (1.8)	10/226 (4.4)		
	Important	609/1770 (34.4)	505/1544 (32.7)	104/226 (46)		
	Indispensable	1123/1770 (63.4)	1011/1544 (65.5)	112/226 (49.6)		
**Petting**						.42
	Unimportant	617/1741 (35.4)	546/1527 (35.8)	71/214 (33.2)		
	Important	887/1741 (50.9)	779/1527 (51)	108/214 (50.5)		
	Indispensable	237/1741 (13.6)	202/1527 (13.2)	35/214 (16.4)		
**The first time**						.01
	Unimportant	333/1768 (18.8)	276/1543 (17.9)	57/225 (25.3)		
	Important	1016/1768 (57.5)	890/1543 (57.7)	126/225 (56)		
	Indispensable	419/1768 (23.7)	377/1543 (24.4)	42/225 (18.7)		
**Contraception**						.02
	Unimportant	165/1737 (9.5)	141/1519 (9.3)	24/218 (11)		
	Important	837/1737 (48.2)	717/1519 (47.2)	120/218 (55)		
	Indispensable	735/1737 (42.3)	661/1519 (43.5)	74/218 (33.9)		
**Emergency contraception**						.01
	Unimportant	136/1739 (7.8)	115/1521 (7.6)	21/218 (9.6)		
	Important	921/1739 (53)	790/1521 (51.9)	131/218 (60.1)		
	Indispensable	682/1739 (39.2)	616/1521 (40.5)	66/218 (30.3)		
**Pregnancy**						.13
	Unimportant	146/1765 (8.3)	121/1542 (7.8)	25/223 (11.2)		
	Important	893/1765 (50.6)	777/1542 (50.4)	116/223 (52)		
	Indispensable	726/1765 (41.1)	644/1542 (41.8)	82/223 (36.8)		
**Abortion**						<.001
	Unimportant	110/1761 (6.2)	85/1540 (5.5)	25/221 (11.3)		
	Important	696/1761 (39.5)	584/1540 (37.9)	112/221 (50.7)		
	Indispensable	955/1761 (54.2)	871/1540 (56.6)	84/221 (38)		
**Sexually transmitted infections**						<.001
	Unimportant	43/1766 (2.4)	29/1542 (1.9)	14/224 (6.3)		
	Important	556/1766 (31.5)	465/1542 (30.2)	91/224 (40.6)		
	Indispensable	1167/1766 (66.1)	1048/1542 (68)	119/224 (53.1)		
**Sexual abuse, harassment, and violence**						<.001
	Unimportant	58/1764 (3.3)	44/1541 (2.9)	14/223 (6.3)		
	Important	460/1764 (26.1)	380/1541 (24.7)	80/223 (35.9)		
	Indispensable	1246/1764 (70.6)	1117/1541 (72.5)	129/223 (57.8)		
**Sex and drugs**						.02
	Unimportant	240/1761 (13.6)	199/1543 (12.9)	41/218 (18.8)		
	Important	809/1761 (45.9)	707/1543 (45.8)	102/218 (46.8)		
	Indispensable	712/1761 (40.4)	637/1543 (41.3)	75/218 (34.4)		
**Cybersex**						.58
	Unimportant	721/1759 (41)	626/1540 (40.6)	95/219 (43.4)		
	Important	788/1759 (44.8)	697/1540 (45.3)	91/219 (41.6)		
	Indispensable	250/1759 (14.2)	217/1540 (14.1)	33/219 (15.1)		
**Cyberbullying**						<.001
	Unimportant	206/1749 (11.8)	157/1531 (10.3)	49/218 (22.5)		
	Important	746/1749 (42.7)	645/1531 (42.1)	101/218 (46.3)		
	Indispensable	797/1749 (45.6)	729/1531 (47.6)	68/218 (31.2)		

^a^Calculated from chi-squared tests.

### Variables Related to Social Media

Of the entire sample, 90.7% (n=1537) of respondents do not visit other affective-sexual education websites. Among the participants who answered the question about which affective sexual education website they usually consult, the website Adolescents [22] scored the highest, at 21.2% (24/113; see the description of the Adolescents webpage in the discussion section). On the other hand, the influencer that respondents most often reported following for the topic of sexuality was Noemí Casquet (@mamacasquet). Finally, it should be noted that 70.5% (n=1200) of the young people in this study reported that they visit pornographic websites (Table 2).

## Discussion

### Sociodemographic Variables

The study is an updated snapshot of ASGD [1] throughout the entire region of Catalonia. In 2021, a report was presented on sexual and reproductive health and rights by the Public Health Agency of Barcelona (ASPB) [2]. The data described in it only represent the population of Barcelona, while the sample in this study includes all the territorial management of Catalonia. In addition, the ASPB report does not distinguish between biological sex and gender identity. The data are displayed in a graph with a completely binary presentation of gender identity (male or female). For these reasons, the data from that study cannot be compared with our findings. Despite all this, it should be noted that in our study, 70% (943/1347) of people with female biological sex affirmed that they were heterosexual, as did 82.62% (352/426) of people with male biological, whereas in the ASPB document data, 87.7% (1850/2110) of female respondents said they were heterosexual, as did 91.8% (1735/1890) of male respondents.

### Variables Related to the WSJ

This study evaluates the WSJ, much like other authors have done with the affective sexual education websites in their countries. The Sexunzipped website for the United Kingdom population over the age of 16 years is one example of several affective sexual education websites that have been created and evaluated through research projects [23-26]. In the last 5 years, Bailey’s group has evaluated interventions using mobile text messages, tablets in the waiting rooms of sexual health services, and social networks [27,28].

Of all the content on the WSJ, participants deemed the topic of sexual abuse, harassment, and violence the most indispensable, with a score of 70.6% (n=1246). This is not surprising given that gender-based violence is a widespread problem [29]. Violence against women by their intimate partners and others is currently considered a major public health problem [5]. In Spain, the number of complaints against minors due to gender violence tripled between 2008 and 2017 [30]. Unfortunately, sexist attitudes within couples persist in younger generations. An astonishing 27% of young people in Spain believe that gender violence is normal within couples, and over 80% reported being aware of acts of abuse in couples in their age range [30]. Data on sexual assault not perpetrated by intimate partners from 15 years of age show that between 0.3% and 12% of women have experienced some act of sexual violence [31]. Furthermore, studies have shown that the highest risk of sexual violence occurs during adolescence and young adulthood [4].

### Variables Related to Social Media

The results of this study show that 90.7% (n=1537) of participants did not consult websites and that 78% (n=1322) did not follow influencers specialized in affective sexual education. These figures seem paradoxical considering that 70.5% (n=1200) of young people have seen pornography at some point. Several studies and scoping reviews on the subject warn that watching mainstream pornography influences the sexual and reproductive health of adolescents and young adults [29,32]. They describe it as a serious problem that may increase gender inequality, given the sexual misrepresentation of women. In another study conducted in Spain on the factors related to gender-based violence, it was shown how adolescents aged 12 to 16 years present romantic love myths, sexist attitudes, and sexual double standards, as evident patterns in adolescents with male gender identity [33]. Therefore, as the same study points out, comprehensive affective sexual education is necessary from the earliest ages of childhood, rather than waiting until the age of 16 years, as is the case in Spain [33].

The results indicating scarce use of websites and digital resources on affective sexual education in young people in Catalonia are very different from those obtained in a German study, in which 80% of participants did use such resources [12]. There are significant differences in the motivation for learning about sexual health between the youth in these 2 countries (Germany and Spain) via the internet since the German study explained that they had an overwhelming participation rate [12]. That was not the case in our study, in which it was difficult to motivate young people to answer the questionnaire.

Adolescents [22] is a portal for young people to find everything that interests them, from information about their idols to tips to answer doubts and concerns, quizzes, news, viral videos, gossip, notes, papers, exam summaries, advice on sexuality and relationships, community, etc. This website is advertised to public and private companies as a means of business to obtain information from the target population that uses it. This is not a public health resource for the emotional and sexual education of young people in Catalonia. It is an audiovisual communication business initiative.

One strength of this study which we have not seen in other cross-sectional studies on the subject is the analysis, which takes into account the various subpopulations that compose the full spectrum of ASGD [1].

### Limitations

Often, young people find it difficult to make commitments, and thus effective recruitment becomes more complex. Double consent, required of people under the age of 16 years in Spain, demands more resources and further hinders participation.

From the start of data collection, it was clear that the sample had a high percentage of women. To correct this, other sample collection spaces that were more regularly frequented by men were sought out and the sample was recalculated. Even so, there was much higher participation of women. The low participation of men can be explained by sex and gender stereotypes and roles. Young men have little interest in taking the initiative of controlling their own offspring, delegating this task to their women partners by omission. Consequently, young women visit the ASSIR units much more frequently, consult more affective sexual education websites, and follow more influencers on the subject than young men [12,34].

### Conclusions

The results of this study show that young people are demanding an affective sexual education that pivots on positive sexuality, which is understood as something to be enjoyed, not centered around fear, and removed from the coitocentric, biomedical, heteropatriarchal, and binary model. This type of sexuality is based on self-esteem and self-care, as well as respect and empathy.

The WSJ, as the digital resource on affective sexual education that it is, must undergo a reformulation process to become a tool for this positive sexuality, with foundations in ASGD.

## Appendix

Multimedia Appendix 1 Questionnaire.

## Abbreviations

AQUAS: Agency of Health Quality and Assessment of Catalonia
ASGD: affective-sexual and gender diversity
ASPB: Public Health Agency of Barcelona
ASSIR: Atenció a la Salut Sexual i Reproductiva
ICS: Institut Català de la Salut
STI: sexually transmitted infection
STROBE: Strengthening the Reporting of Observational Studies in Epidemiology
TSI: Territorial Socioeconomic Index
WSJ: Website Sexe Joves

## Data Availability

The data that support the findings of this study are available on request from the corresponding author. The data are not publicly available due to privacy or ethical restrictions.
