# Supplementary material for: Adolescents and Young Adults Evaluating a Website for Affective-Sexual Information and Education: Multicenter Cross-Sectional Study
Source: J Med Internet Res. 2023 Oct 26;25:e49962. doi: 10.2196/49962 (PMC10636610; doi:10.2196/49962)
Supplement: Multimedia Appendix 1 [file jmir_v25i1e49962_app1.docx]

**Figure 1.** Questionnaire

### Where did you get this questionnaire?

- An educational centre
- A health centre
- Other

### Year of birth

### Sex

- Penis
- Vulva
- Intersex

### Sexual orientation

- Heterosexual
- Bisexual
- Homosexual
- Other

### Gender identity

- Man
- Woman
- Non-binary
- Other

### Country of origin of your parents/legal guardians (if they are from different countries, write both and indicate who is from where. E.g.: Colombia: mother; Argentina: father)

### How many years have you lived in Spain?

### City/town where you live

### Postal code

### Ongoing education

- I don't study
- Secondary school
- High school
- Intermediate level vocational training
- Higher level vocational training
- Training and insertion programs
- University

### Do you have a job?

- Yes
- No

### Are you familiar with the website Sexe Joves?

- Yes
- No

### Do you think the young people in your milieu know of it?

- Yes
- No

### Do you use it?

- Yes
- No

### Only answer if you do use it. From what device do you use it?

- Mobile phone
- Tablet
- Computer

### Only answer if you do use it. Is it easy to access the content you are looking for?

- Yes
- No

### Only answer if you do use it. Do you find it useful?

- Yes
- No

### Only answer if you do use it. What do you use most? (You may tick more than one option.)

- Content
- Chat
- Email

### Do you visit other websites about affective-sexual education?

- Yes
- No

### Which ones?

### Do you follow any influencers who talk about affective-sexual education?

- Yes
- No

### Could you tell us their names?

### Have you ever visited porn websites?

- Yes
- No

### Rank the following content in order of importance to you.

Unimportant Important Indispensable

|  |
| --- |
|  |
|  |
|  |
|  |
|  |
|  |
|  |
|  |
|  |
|  |
|  |
|  |

|  |
| --- |
|  |
|  |
|  |
|  |
|  |
|  |
|  |
|  |
|  |
|  |
|  |
|  |

|  |
| --- |
|  |
|  |
|  |
|  |
|  |
|  |
|  |
|  |
|  |
|  |
|  |
|  |

Emotion and sexuality

Knowledge of your body

Petting

The first time

Contraception

Emergency contraception

Pregnancy

Abortion

Sexually transmitted infections

Abuse, harassment and sexual violence

Sex and drugs

Cybersex

Cyberbullying
